# Effects of sleep self-monitoring via app on subjective sleep markers in student athletes

**DOI:** 10.1007/s11818-022-00395-z

**Published:** 2022-10-25

**Authors:** Sarah Jakowski, Moritz Stork

**Affiliations:** Faculty of Sport Science, Ruhr University Bochum, Gesundheitscampus Nord 10, 44801 Bochum, Germany

**Keywords:** Sleep monitoring, Self-assessment, Sleep quality, Sleep behaviour, Bedtime procrastination, Schlafmonitoring, Selbstvermessung, Schlafqualität, Schlafverhalten, Schlafprokrastination

## Abstract

As sleep problems are highly prevalent among university students and competitive athletes, and the application of commercial sleep technologies may be either useful or harmful, this study investigated the effects of a 2-week sleep self-monitoring on the sleep of physically active university students (*n* *=* 98, 21 ± 1.7 years). Two intervention groups used a free sleep app (*Sleep Score*; SleepScore Labs™, Carlsbad, CA, USA: *n* = 20 or *Sleep Cycle*; Sleep Cycle AB, Gothenburg, Sweden: *n* = 24) while answering online sleep diaries. They used the app analysis function in week 1 and the ‘smart alarm’ additionally in week 2. As controls, one group answered the online sleep diary without intervention (*n* *=* 21) and another the pre–post questionnaires only (*n* *=* 33). Facets of subjective sleep behaviour and the role of bedtime procrastination were analysed. Multilevel models did not show significant interactions, indicating intervention effects equal for both app groups. Sleep Cycle users showed trends toward negative changes in sleep behaviour, while the online sleep diary group showed more, tendentially positive, developments. Bedtime procrastination was a significant predictor of several variables of sleep behaviour and quality. The results indicate neither benefits nor negative effects of app-based sleep self-tracking. Thus, student athletes do not seem to be as susceptible to non-validated sleep technologies as expected. However, bedtime procrastination was correlated with poor sleep quality and should be addressed in sleep intervention programmes.

## Abstract (de)

Da Schlafprobleme unter Studierenden und im Leistungssport verbreitet sind und die Anwendung kommerzieller Schlaftechnologien potenziell nützlich, aber auch schädlich sein kann, wurden in dieser Studie die Effekte eines 2‑wöchigen Schlaf-Selbstmonitorings auf das Schlafverhalten von sportlich aktiven Studierenden untersucht (*n* *=* 98, 21 ± 1,7 Jahre). Dazu nutzten 2 Interventionsgruppen neben einem Online-Schlafprotokoll eine kostenlose Schlaf-App (*Sleep Score*, Fa. SleepScore Labs™, Carlsbad/CA, USA: *n* *=* 20; *Sleep Cycle*, Fa. Sleep Cycle AB, Göteborg, Schweden: *n* *=* 24). In Woche 1 nutzten sie die Analysefunktion und in Woche 2 zusätzlich die „intelligente“ Weckfunktion. Zur Kontrolle füllte eine andere Gruppe nur Online-Schlafprotokolle (*n* *=* 21) und eine weitere nur die Prä-post-Befragung (*n* *=* 33) aus. Analysiert wurden Facetten des subjektiven Schlafverhaltens sowie die Rolle der Schlafprokrastination. Insgesamt zeigten Mehrebenenmodelle kaum signifikante Interaktionseffekte, die auf Interventionseffekte in beiden App-Gruppen hindeuten. Anwendende von Sleep Cycle zeigten tendenziell negative Veränderungen im Schlafverhalten, während die Schlafprotokollgruppe die meisten, tendenziell positiven Veränderungen aufwies. Schlafprokrastination erwies sich zudem als signifikanter Prädiktor für viele Variablen des Schlafverhaltens und der Schlafqualität. Die Befunde deuten weder auf einen Nutzen noch auf negative Effekte durch App-basierte Schlaf-Selbstvermessung hin. Sportlich aktive Studierende scheinen daher nicht so anfällig für nichtvalidierte Schlaftechnologien zu sein wie erwartet. Allerdings hängt Schlafprokrastination mit geringerer Schlafqualität zusammen und sollte daher Ziel von Interventionsprogrammen sein.

As sleep problems are highly prevalent among athletes and university students, commercial sleep technologies may represent low-threshold applications to track and optimise sleep. However, sleep self-tracking may either enhance sleep behaviour through supporting self-monitoring, or exacerbate sleep through inaccurate feedback that leads to obsessive preoccupation with one’s sleep. Thus, the effects of smartphone sleep apps need to be examined. Moreover, postponing bedtime without obvious reason (bedtime procrastination) is a common phenomenon among young people, which requires further consideration.

Sleep problems in young adults, specifically university students, are very common [26, 29]. Furthermore, competitive athletes constitute a vulnerable population with poor sleep quality and/or quantity [12, 24, 28]. External factors (e.g. academic or social demands [29], training and competitions [5, 24]), but also lifestyle, behavioural and psychological factors affect sleep [29]. For instance, self-regulation plays an important role in terms of regular, healthy sleep patterns [17]. The phenomenon of postponing bedtime without external reasons (i.e. bedtime procrastination [BP]) is associated with negative consequences for sleep duration and daytime functioning [22]. University students seem to present higher manifestations of BP than the general population [14], while there are currently no data for athletes.

It was assumed that athletes and university students are highly interested in monitoring and optimising their sleep. Commercial sleep technologies (CST; e.g. wearables, smartphone apps) are low-threshold options to track and manage aspects of sleep. Proprietary sensors of smartphones assess movements and sounds which generate estimations of sleep duration and sleep quality [21]. They provide feedback on the previous night’s sleep and may include a ‘smart alarm’ which is supposed to enhance a smooth and refreshed awakening. However, CST are criticised due to their lack of validity and unfathomable algorithms [4, 7]. Usually, data reports are limited to graphical representations instead of standardised sleep parameters [11]. Thus, CST either underestimate or overrate sleep [30]. This inaccurate or false feedback may have severe consequences, as it either conceals manifest sleep disorders or misdiagnoses a sleep disorder, which encourages dysfunctional behavioural adaptations [30]. Baron et al. [2] postulate that even the mere measurement of sleep via CST may lead to problematic behaviours and an obsessive preoccupation with sleep optimisation. However, there is limited evidence on the actual effects of using CST on sleep parameters and subjective perceptions. The assumed susceptibility of student athletes also requires further examination. Thus, the aim of this study was to investigate the effects of a 2-week intervention on subjective sleep behaviour and the role of BP among physically active university students. The first aim was to analyse the participants’ evaluation of two different smartphone apps after using the sleep tracking as well as the ‘smart alarm’ feature. The second aim was to analyse the effects of the smartphone apps on (a) subjective sleep quality, (b) selected sleep parameters and (c) daily sleep patterns. In addition, the role of BP among these effects was examined.

## Methods

### Study design and procedure

The study took place online in November and December 2020. Participants were recruited via classes for physical education and psychology students who could collect experimental credits. Four groups were assigned in a randomised controlled way based on the e‑mail addresses that were provided in a random order separately from the survey data. Control group 1 (CG1) answered only the pre and post questionnaires 2 weeks apart, while control group 2 (CG2) and the intervention groups (IG) filled in online sleep diaries (OSD) every morning and evening for 2 weeks. CG2 did not receive further instructions. IG1 was invited to install the app *Sleep Score* (SleepScore Labs™, Carlsbad, CA, USA) and IG2 *Sleep Cycle* (Sleep Cycle AB, Gothenburg, Sweden) for free.

In week 1 (W1), intervention group participants used the sleep tracking feature and observed the apps’ reports, while in W2, participants additionally used the ‘smart alarm’. *Sleep Score* assesses body movements and respiration rates via the smartphone’s acceleration sensors and microphone. The sleep tracking feature calculates a score from 0 to 100, with 100 indicating optimal sleep quantity and quality. Continuity and duration of the sleep stages are also reported. With regular application, a graphical representation of the data can be obtained. The ‘smart alarm’ is activated by indicating the final wake time and a timeframe in which to be woken up. Using the sleep tracking feature, the app detects light sleep within this timespan and initiates the alarm. *Sleep Cycle* works in a similar way, yet without generating an overall sleep score. Instead, it provides more graphical analyses of sleep parameters. *Sleep Cycle* was shown to perform poorly in comparison to PSG; therefore, the app data were not used in the statistical analyses [7, 11].

### Participants

Initially, 141 participants were recruited. Only those who answered the pre–post questionnaires and those in IG1 and IG2 who completed the OSD on at least 5 days during W2 were included in the analysis. Thus, the final sample consisted of 98 participants (*n* = 61 female; mean 21 ± 1.7 years, range 18–28 years). Regular physical activity was reported on 5.8 ± 3.6 d/week, with a mean duration of 6.9 ± 5.3 h/week. The majority participated in individual sports (*n* = 71) and 50% competed regularly in competitions (*n* = 49). The final groups consisted of *n* = 33 (CG1), *n* = 21 (CG2), *n* = 20 (IG1), and *n* = 24 (IG2).

Informed consent was obtained in the pre-study questionnaire and only those who consented to participate voluntarily could continue the survey. Individual feedback was provided to interested participants. Ethical clearance was obtained from the faculty’s local ethics committee prior to the start of the study.

### Instruments

#### Sleep app evaluation

IG1 and IG2 rated their expectations and experiences with the respective app. Specifically, they rated (a) whether the tracking feature resembled their own sleep perception, (b) whether they considered the app’s results as reliable, (c) whether the ‘smart alarm’ indeed chose the optimal timing, (d) whether they felt more refreshed upon waking compared to an ordinary alarm and whether they were willing to continue using (e) sleep tracking as well as (f) ‘smart alarm’. Furthermore, they judged (g) whether they consider sleep self-tracking via app as useful, and (h) whether they used the app’s data to optimise their sleep behaviour. These items were assessed on a scale ranging from 0 (not at all) to 4 (totally). Finally, participants gave (i) an overall evaluation of their app experience from 0 (very negative) to 4 (very positive).

#### Pittsburgh Sleep Quality Index

The pre–post questionnaires contained the *Pittsburgh Sleep Quality Index* (PSQI) to assess sleep quality [6]. Hereby, 19 items are summarised to seven components with scores ranging from 0 to 3 (i.e. subjective sleep quality, sleep latency, sleep duration, sleep efficiency, sleep disorders, sleep medication and daytime sleepiness). The sum of these component scores generates the total score (0–21). Scores ≤ 5 indicate good sleep quality [6, 15]. While the original version covers the previous 4 weeks, the current study used a 2-week timeframe. The applicability of this modification has been shown previously [1]. Internal consistency in this sample was Cronbach’s α = 0.60 at pre. In addition, information on bedtime, get-up time and sleep onset latency was used to calculate the sleep parameters “total sleep time” (TST) and “sleep efficiency” (SE; relation of time spent in bed to TST), which were integrated in the pre–post analyses.

#### Bedtime Procrastination Scale

At the pre and post timepoints, the nine-item *Bedtime Procrastination Scale* (BPS) was used to assess bedtime procrastination [22]. The frequency of postponing bedtime is rated on a scale ranging from 1 ([almost] never) to 5 ([almost] always). The mean value represents the BPS score, with higher values indicating a higher prevalence of BP. Internal consistency was Cronbach’s α = 0.88 at pre.

#### Online sleep diary

The OSD consisted of an adapted evening and morning protocol according to Hoffmann et al. [18]. Before going to bed, daytime activities and mood states were documented, as was the planned bedtime. Within 30 min of getting up, participants rated restfulness and the events of last night’s sleep. Specifically, they documented the actual time of lights out, when they awoke, and when they finally got up in the morning. They estimated sleep onset latency and the frequency and duration of awakenings. Using these variables, the sleep parameters “time in bed” (TIB), TST and SE were calculated. The difference (in minutes) between planned bedtime and actual time of lights out was used as an indicator of daily bedtime procrastination (DBP).

### Statistical analyses

Data preparation and descriptive and statistical analyses were performed with Microsoft Excel 2019 (Microsoft Corporation, Redmond, WA, USA), the statistical software SPSS (version 26, IBM Corp., Armonk, NY, USA) and RStudio (version 4.1.2, PBC, Boston, MA, USA).

It must be mentioned that different features and functions of *Sleep Score* (IG1) between Android and iOS smartphone systems became apparent only during the data collection. Android users received more limited feedback compared to iOS users. In IG2, *n* = 16 were iOS and *n* = 5 were Android users. Thus, in a preliminary step, the effect of smartphone system (SYS) on the subjective evaluation of the app was analysed. Within IG2 (*Sleep Cycle*), *n* = 11 were iOS and *n* = 12 were Android users. To examine the first study aim, moderated regression analyses were conducted with the items of the app evaluation as dependent variables. Dummy-coded variables app (0 = *Sleep Score*, 1 = *Sleep Cycle*) and SYS (0 = iOS, 1 = Android) served as predictors, and expectations at pre (item g and h) as covariates.

Since some effects of SYS were identified, this variable was included in subsequent analyses. The second study aim was investigated via linear mixed models with random intercepts using the maximum likelihood method [10]. Timepoints served as level 1 (pre = 0 vs. post = 1, W1 = 0 vs. W2 = 1) and individuals as level 2 predictors. Dependent variables were PSQI score, pre–post sleep parameters (TST, SE) and OSD parameters (TIB, TST, SE, DBP). Intercept-only models were calculated first (M0). Subsequent models included time (M1), group*time interaction with CG1 as the reference group (M2) and SYS (M3) as predictors. As the information about the system was obtained only for IG1 and IG2, this variable was coded as ‘unknown’ in CG1 and CG2, which also served as the reference group. M4 included the grand mean of the centred BPS pre score as a covariate. As M3 did not show significant improvements to the model, the more parsimonious M2 was chosen for building M4. For each model, intraclass correlation coefficients (ICC) were determined to show the relation between inter- and intraindividual variance. Descriptive model parameters, i.e. the Akaike information criterion (AIC) and Bayesian information criterion (BIC) as well as chi^2^ likelihood ratio tests, were used to compare nested models. However, in case of missing data for single variables, direct model comparisons were not possible, as those models were not considered as nested. The significance of single fixed effects coefficients was tested via Satterthwaite-approximated *t*-tests. The analyses were conducted with the lme4 package [3]. Level of significance was set to *p* < 0.05.

## Results

### Subjective app evaluation

**Fig. 1:**
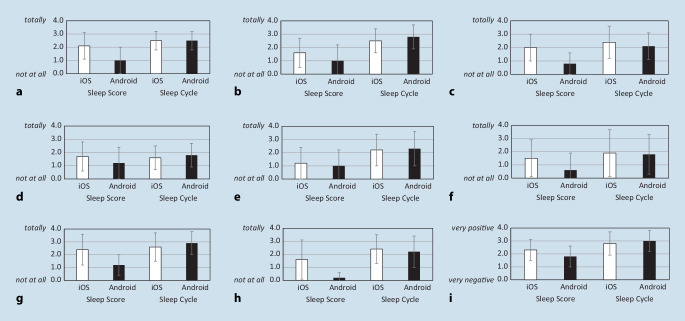
Distribution of the evaluation ratings of the two apps. **a** Perceived agreement. **b** Perceived reliability. **c** Optimal timing of ‘smart alarm’. **d** More refreshed awakening. **e** Continue use of tracking feature. **f** Continue use of ‘smart alarm’. **g** Usefulness of sleep tracking app. **h** Optimised sleep behaviour. **i** Overall evaluation

Fig. 1 shows the distribution of the app evaluations by IG1 and IG2 separated for SYS. It seems that participants of IG1 gave lower ratings irrespectively of SYS, as scores did not exceed values > 2.5. An effect was found in (a) perceived agreement only for SYS. Android users tended to disagree more often (*b* = −1.13, *p* = 0.014). The interaction with group was not significant (*b* = 1.08, *p* = 0.064). There was also a significant effect for (b) indicating that IG2 rated *Sleep Cycle* as more reliable than IG1 did *Sleep Score* (*b* = 0.98, *p* = 0.017). Although the regression analysis was not significant (Table 1), SYS significantly predicted the rating (c) whether the ‘smart alarm’ woke at an optimal time (*b* = −1.20, *p* = 0.032), indicating higher disagreement among Android users. In terms of (d) feeling more refreshed in the morning, the regression was not significant. Another significant effect was identified for (e) probably continue sleep tracking with the app, with IG2 showing higher consent (*b* = 0.99, *p* = 0.046). However, the regression of (f) probably continuing the ‘smart alarm’ was not significant (Table 1). Moreover, the rating of (g) the usefulness of using the app was significantly higher for iOS users (*b* = −3.21, *p* = 0.013), while IG2 showed generally higher scores on a descriptive level. No significant effect was found for (h) whether participants used the app to optimise their sleep behaviour. Finally, the overall evaluation revealed a main effect for SYS with higher ratings for iOS users (*b* = −1.51, *p* = 0.001) and an interaction with group which indicated that Android users rated *Sleep Score* (IG1) more negatively (*b* = 1.69, *p* = 0.004). Overall, the results not only show that both apps were rated differently, but also that different features for Android users seem to be responsible for the ratings.

**Table 1:** Results of subjective app evaluations

	Regression analysis			
	*F*	*p*-value	*R*^*2*^	Adjusted *R*^*2*^
a) Perceived agreement^a^	4.38	0.009	0.247	0.191
b) Perceived reliability^a^	3.40	0.002	0.304	0.252
c) Optimal timing of ‘smart alarm’^a^	2.63	0.064	0.165	0.102
d) More refreshed awakening^a^	0.38	0.768	0.028	−0.045
e) Continue use of tracking feature^a^	3.07	0.039	0.187	0.126
f) Continue use of ‘smart alarm’^a^	0.93	0.436	0.065	−0.005
g) Usefulness of sleep tracking app^b^	2.45	0.041	0.256	0.210
h) Optimised sleep behaviour^c^	2.23	0.055	0.308	0.170
i) Overall evaluation^a^	9.11	< 0.001	0.406	0.361

^a^Degrees of freedom (*df*) = 3,40

^b^Ratings of the pre questionnaire were included as covariate, *df* = 7, 31

^c^Ratings of the pre questionnaire were included as covariate, *df* = 7, 35

### Online sleep diary

**Fig. 2:**
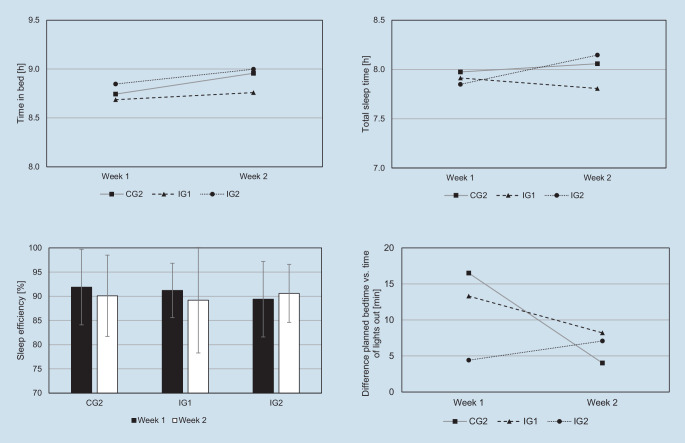
Descriptive values of daily sleep parameters separated by group. *CG2* online sleep diary only, *IG1* app group *Sleep Score*, *IG2* app group *Sleep Cycle*

Fig. 2 presents the descriptive results of TIB, TST, SE and DBP with separate lines for CG2, IG1 and IG2. Descriptive model parameters, chi^2^ likelihood ratio tests and ICCs can be seen in Table 2. Slight increases were observed in TIB for CG2 and IG1 in W2, while values were constantly > 8.5 h in each group. M0 revealed that 19.5% of variance was explained by interindividual and 80.5% by intraindividual differences. There were no significant main or interaction effects in the random-intercept models for this parameter. Average TST was < 8 h in all groups in W1, whereas TST slightly increased in CG2 and IG2 in W2 and even decreased in IG1. ICC indicated that 12.4% of variance was explained by interindividual differences (M0). No significant main or interaction effects were identified in the random-intercept models. On a descriptive level, SE decreased slightly in CG2 and IG1 in W2, while SE slightly increased in IG2 (Fig. 2). In M0, 15.5% of variance was explained by interindividual differences. Adding the time variable in M1 did not yield any significant effects, whereas M2 slightly improved (*p* = 0.072, Table 2). The time effect was not significant (*b* = −1.60, *p* = 0.082), but there was a main effect for IG2 indicating lower SE compared to CG2 (*b* = −2.47, *p* = 0.044). Moreover, the time*group interaction was significant for IG2, indicating higher SE in W2 (*b* = 2.60, *p* = 0.042). Controlling for SYS (M3) and BP (M4) did not reveal further significant effects. Regarding DBP, CG2 and IG1 showed tendencies towards reduction and IG2 an increased value in W2 (Fig. 2). M0 revealed that 12.3% of variance was explained by interindividual differences (Table 2). M1 yielded no significant time effects, which were only tendentially identified in M2 (*b* = −11.59, *p* = 0.075). Controlling for SYS did not provide significant effects (M3). However, BP was identified as a significant predictor in M4 (*b* = 11.30, *p* = 0.002), indicating that higher scores of BPS are associated with higher differences between planned bedtime and actual time of lights out.

**Table 2:** Model parameters of daily sleep parameters and pre–post measurements

	ICC (adjusted)	AIC	BIC	Comparison of nested models
**Daily sleep parameters**				
*Time in bed*				
M0	0.195	14,360.4	14,374.1	N/A
M1	0.195	14,361.2	14,379.6	χ^2^ = 1.15, df = 1, *p* = 0.283
M2	0.195	14,368.4	14,405.1	χ^2^ = 0.77, df = 4, *p* = 0.942
M3	0.182	14,120.4	14,161.5	–
M4	0.179	13,898.9	13,939.9	–
*Total sleep time*				
M0	0.124	14,979.8	14,993.7	N/A
M1	0.125	14,980.5	14,999.0	χ^2^ = 1.26, df = 1, *p* = 0.262
M2	0.123	14,985.5	15,022.5	χ^2^ = 3.01, df = 4, *p* = 0.557
M3	0.117	14,719.7	14,761.2	–
M4	0.110	14,481.0	14,522.3	–
*Sleep efficiency*				
M0	0.155	5191.9	5205.7	N/A
M1	0.156	5191.5	5210.0	χ^2^ = 2.40, df = 1, *p* = 0.121
M2	0.151	5190.9	5227.9	χ^2^ = 8.59, df = 4, *p* = 0.072
M3	0.149	5109.6	5151.1	–
M4	0.145	5021.6	5062.9	–
*Planned bedtime vs. actual time of lights out*				
M0	0.123	7778.1	7791.8	N/A
M1	0.123	7778.5	7796.8	χ^2^ = 1.63, df = 1, *p* = 0.202
M2	0.120	7783.4	7820.1	χ^2^ = 4.65, df = 5, *p* = 0.460
M3	0.118	7657.6	7698.7	–
M4	0.097	7535.2	7576.2	–
**Pre–post measurements**				
*PSQI score*				
M0	0.524	821.5	831.1	N/A
M1	0.546	818.1	830.9	χ^2^ = 5.39, df = 1, *p* = 0.020
M2	0.556	825.5	857.7	χ^2^ = 4.54, df = 6, *p* = 0.605
M3	0.554	826.9	862.3	χ^2^ = 0.68, df = 1, *p* = 0.411
M4	0.529	781.8	816.6	–
*Total sleep time*				
M0	0.588	2106.6	2116.5	N/A
M1	0.590	2108.1	2121.3	χ^2^ = 0.51, df = 1, *p* = 0.476
M2	0.595	2109.5	2142.2	χ^2^ = 10.68, df = 6, *p* = 0.098
M3	0.592	2110.8	2146.8	χ^2^ = 0.69, df = 1, *p* = 0.406
M4	0.595	1891.2	1926.1	–
*Sleep efficiency*				
M0	0.446	1450.0	1459.8	N/A
M1	0.447	1451.9	1465.0	χ^2^ = 0.08, df = 1, *p* = 0.772
M2	0.461	1456.5	1489.3	χ^2^ = 7.34, df = 6, *p* = 0.290
M3	0.461	1458.5	1494.5	χ^2^ = 0.06, df = 1, *p* = 0.806
M4	0.484	1311.0	1346.6	–

*PSQI* Pittsburgh Sleep Quality Index, *ICC* intraclass coefficient, *AIC* Akaike information criterium, *BIC* Bayesian information criterium, *M0* intercept-only model, *M1* time effect, *M2* time*group interaction, *M3* time*group interaction controlled for smartphone system, *M4* time*group interaction controlled for bedtime procrastination, – Model comparison not possible, *N/A* not applicable

### Pre–post analyses of sleep quality and sleep parameters

**Fig. 3:**
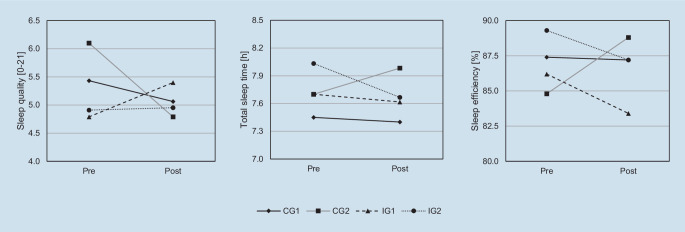
Descriptive values of pre–post sleep parameters separated by group. *CG1* control group 1, *CG2* online sleep diary only, *IG1* app group *Sleep Score*, *IG2* app group *Sleep Cycle*

Descriptive pre–post comparisons of PSQI are depicted in Fig. 3. While CG1 and CG2 showed decreased scores in W2, they increased in IG1 and IG2. Model parameters and ICCs can be seen in Table 2. M0 revealed that 52.4% of variance was explained by interindividual differences. A significant time effect was found in M1 (*b* = −0.56, *p* = 0.021), which significantly improved the model (*p* = 0.020, Table 2). However, upon adding group (M2) and controlling for SYS (M3), no main or interaction effects were identified. Upon adding BP (M4), the time effect was significant (*b* = −0.61, *p* = 0.013) and BPS was a significant predictor indicating poorer sleep quality with higher BPS scores (*b* = 0.83, *p* = 0.003).

Descriptive pre–post comparisons of TST and SE are shown in Fig. 3 and model parameters and ICCs in Table 2. In all groups, TST slightly decreased at post, except for CG2 which showed increased TST. In M0, 58.8% of variance was explained by interindividual differences. While no significant effects were found in M1, a group effect was identified in M2, indicating more TST in IG2 compared to CG1 (*b* = 34.10, *p* = 0.026). Controlling for SYS (M3) did not improve the model (Table 2), but this group effect was stronger (*b* = 40.48, *p* = 0.018). Furthermore, BP was identified as a significant predictor (M4; *b* = −15.71, *p* = 0.018). The group effect for IG2 was slightly poorer (*b* = 24.98, *p* = 0.017), but two time*group interactions were found. IG2 showed decreased TST (*b* = −24.96, *p* = 0.008) and CG2 increased TST (*b* = 25.66, *p* = 0.010) at post when controlled for BP (M4).

On the descriptive level, comparable developments were observed for SE (Fig. 3). M0 indicated that 44.6% of variance was explained by interindividual differences. Consecutive models did not yield significant main or interaction effects except for M4. Only the time*group interaction for CG2 was significant, indicating higher SE at post (*b* = 5.57, *p* = 0.008).

## Discussion

The aim of this study was to analyse the effects of a 2-week sleep self-tracking intervention using two commercial smartphone apps. According to the subjective evaluation of the apps, it seems that, overall, participants did not benefit from a ‘successful’ sleep management intervention. There was generally low agreement between perceived sleep and the apps’ feedback. As 19.1% of the explained variance indicates, other factors seem to determine the perceived match of sleep assessments. Moreover, different features of *Sleep Score* for Android and iOS users led to diverse evaluations. In terms of perceived reliability of the apps, SYS was not identified as predictor. However, *Sleep Cycle* (IG2) was rated as more reliable than *Sleep Score *(IG1), which was also supported by higher ratings of continuing to use the sleep tracking feature. Overall, IG2 evaluated *Sleep Cycle* more positively than IG1 rated *Sleep Score*. However, the ‘smart alarm’ was not perceived as effective. These results underscore the fact that the application of CST is more complex than assumed. Different providers and features need to be carefully considered, as they may influence the results. As participants received credit points for participation, intrinsic motives for their participation were not known. Roomkham et al. [25] identified five motive styles of engaging in self-tracking, i.e. directive, documentary, diagnostic, collection rewards, and fetishized tracking. Future studies may consider participants’ needs and elaborate distinguished patterns of possible effects.

Analyses of daily sleep behaviour also did not indicate definite effects of the sleep apps. Participants did not report staying longer/for less time in bed or obtaining more/less sleep. Notably, mean TST was > 7 h in all groups, so participants seem to get sufficient sleep [16]. Regarding DBP, the findings highlight that this is a rather intraindividual phenomenon that is not influenced by using a ‘smart alarm’. Nevertheless, controlling for BP revealed that the difference between planned and actual bedtime was slightly reduced in W2. As higher scores are associated with higher differences, findings support the theoretical assumption of Kroese et al. [22]. It has to be mentioned, though, that the construct may have been inaccurately captured, as the evening item asked for planned bedtime and the morning item for actual time of lights out. Future studies should concretise the meaning of bedtime (going to bed) and shuteye-time (intending to sleep) [8]. It may also be useful to distinguish between bedtime procrastination and while-in-bed procrastination [23].

Considering the pre–post analyses including control group 1, no effect was identified for either app group. Notably, CG2 showed the highest decrease of the PSQI score descriptively, while IG1 showed higher scores, just above the cut-off. Remarkable was that upon adding BPS to the model, the time effect became stronger, supporting the assumption that higher BP is associated with reduced sleep quality. A similar effect of BP was observed for TST. In addition, IG2 reported less and CG2 more TST at post compared to CG1, which was also comparable for SE. However, as the timeframe was 2 weeks and sleep was actively influenced during week 2 only, definite intervention effects were probably difficult to detect [1]. The somewhat poor reliability of the PSQI in this sample might also have impaired the findings.

One major limitation of the study was the lack of objective sleep assessment. Furthermore, the study took place in autumn 2020, when the developments of the coronavirus disease 2019 pandemic were unpredictable. As classes took place online, there was no direct contact with the participants. The pandemic was also a limiting factor for sports activities, as participants were not able to train and perform in the usual way. This may also have affected social life and sleep behaviour. General fitness and training volume significantly decreased during lockdown regulations, which negatively affected sleep quality and negative emotions [20]. Interestingly, positive effects on sleep regularity and quantity were found for professional and semi-professional athletes [9]. It was assumed that athletes might have gained more interest in analysing and optimising their sleep during that period. However, the present findings do not support this assumption, which is also in line with another survey [19]. Moreover, the sample of student athletes may not be representative for youth athletes, among whom external factors (e.g. late training, homework) and later circadian sleep drive may be more responsible for delayed bedtime rather than a lack of self-regulation.

Overall, this was the first study to examine the effects of sleep self-tracking via two smartphone apps among student athletes. The present study does not provide evidence for the concerns that using CST exacerbates sleep and increases dysfunctional occupation with one’s sleep [2, 27]. At the same time, potential beneficial effects that were presumed through constructive sleep self-monitoring [7] were also not detected. In conclusion, it seems that student athletes are not as vulnerable and suggestable to sleep self-tracking as assumed [19]. Nevertheless, practitioners and athletes should draw on standardised sleep measures to monitor training and sleep [13], and address adequate sleep hygiene behaviour in order to support performance enhancement and health management.

## Conclusion and implications

- Although university students and athletes are a vulnerable group in terms of experiencing poor sleep, they are not as susceptible to possible inaccurate commercial sleep technologies as was anticipated.
- Conducting sleep self-tracking via smartphone app for 1 week and using the app’s ‘smart alarm’ additionally for another week does not have negative or positive effects on subjective sleep parameters in student athletes.
- Nevertheless, athletes, coaches and consultants should be careful when applying unvalidated technologies and rather rely on standardised empirical methods for sleep monitoring.
- As bedtime procrastination was identified as a predictor of poor sleep quality, interventions should target sleep hygiene behaviour fostering ‘offline’ bed routines and support self-regulation in young active people.

## Declarations

For this article no studies with human participants or animals were performed by any of the authors. All studies mentioned were in accordance with the ethical standards indicated in each case.
